# An appraisal of the SD_IR_ as an estimate of true individual differences in training responsiveness in parallel‐arm exercise randomized controlled trials

**DOI:** 10.14814/phy2.14163

**Published:** 2019-07-19

**Authors:** Jacob T. Bonafiglia, Andrea M. Brennan, Robert Ross, Brendon J. Gurd

**Affiliations:** ^1^ School of Kinesiology and Health Studies Queen’s University Kingston Ontario

**Keywords:** Individual responses, individual variability, exercise training, SD_IR_

## Abstract

Calculating the standard deviation of individual responses (SD_IR_) is recommended for estimating the magnitude of individual differences in training responsiveness in parallel‐arm exercise randomized controlled trials (RCTs). The purpose of this review article is to discuss potential limitations of parallel‐arm exercise RCTs that may confound/complicate the interpretation of the SD_IR_. To provide context for this discussion, we define the sources of variation that contribute to variability in the observed responses to exercise training and review the assumptions that underlie the interpretation of SD_IR_ as a reflection of true individual differences in training responsiveness. This review also contains two novel analyses: (1) we demonstrate differences in variability in changes in diet and physical activity habits across an intervention period in both exercise and control groups, and (2) we examined participant dropout data from six RCTs and found that significantly (*P* < 0.001) more participants in control groups (12.8%) dropped out due to dissatisfaction with group assignment compared to exercise groups (3.4%). These novel analyses raise the possibility that the magnitude of within‐subject variability may not be equal between exercise and control groups. Overall, this review highlights that potential limitations of parallel‐arm exercise RCTs can violate the underlying assumptions of the SD_IR_ and suggests that these limitations should be considered when interpreting the SD_IR_ as an estimate of true individual differences in training responsiveness.

## Introduction

**Figure 1:**
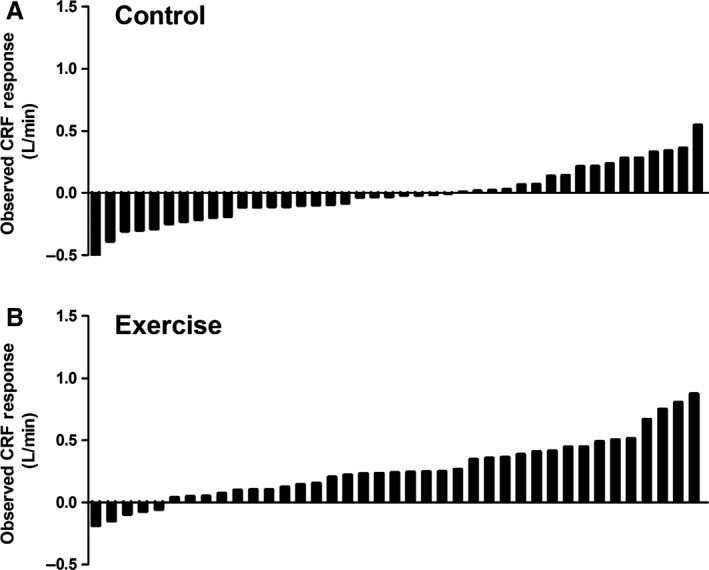
‘Classic’ illustration of variability in the observed responses to exercise training. Individual bars represent observed changes in cardiorespiratory fitness (CRF) for individual participants from a previously published randomized controlled trial (Ross et al., 2015). Observed responses to 24 weeks of a no‐exercise control period (A) or exercise training (B). The exercise training prescription was walking/jogging five times per week at an intensity of 50% baseline cardiorespiratory fitness until 180 (females) or 300 (males) kilocalories were expended.

In 1999 Bouchard et al. (1999) published results from the HERITAGE Family Study demonstrating a wide range of peak oxygen consumption (*V*O_2_peak) responses across individuals completing an identical exercise training program. Subsequently, a substantial body of literature has emerged reporting variability in the observed pre–post training changes in *V*O_2_peak (Hautala et al., 2006; Vollaard et al., 2009; Sisson et al., 2009; Astorino and Schubert, 2014; Wolpern et al., 2015; Ross et al., 2015; Raleigh et al., 2016; Gurd et al., 2016; Bonafiglia et al., 2016; Montero and Lundby, 2017), peak work rate (Vollaard et al., 2009; Montero and Lundby, 2017), lactate threshold (Gurd et al., 2016; Bonafiglia et al., 2016), and other physiologically meaningful central (MacPherson et al., 2011; Astorino et al., 2016; Raleigh et al., 2018) and peripheral (Vollaard et al., 2009; McPhee et al., 2011; Edgett et al., 2016; Bonafiglia et al., 2017; deLannoy et al., 2017; Raleigh et al., 2018) adaptations. Importantly, although the existence of variability in the observed response to training cannot be questioned (illustrated in Figure 1), it remains unclear whether this variability can be attributed to an effect of exercise *per se.*

In the last several years, biostatisticians in the field of exercise science have raised concerns regarding the experimental and statistical rigor required to appropriately analyze individual response heterogeneity (Atkinson and Batterham, 2015; Hopkins, 2015; Hecksteden et al., 2015; Ross et al., 2019; Atkinson et al., 2019). Specifically, although many reports have assumed that the variability in observed responses reflects true individual differences in training responsiveness (Hautala et al., 2006; Vollaard et al., 2009; Sisson et al., 2009; Astorino and Schubert, 2014; Wolpern et al., 2015; Ross et al., 2015; Raleigh et al., 2016; Gurd et al., 2016; Bonafiglia et al., 2016; Montero and Lundby, 2017), recent reviews have highlighted the importance of considering multiple sources of variation that can contribute to the observed variability in training responses and have questioned whether the existence of individual variability attributable to exercise has been convincingly demonstrated (Atkinson and Batterham, 2015; Hopkins, 2015; Hecksteden et al., 2015; Williamson et al., 2017; Hopkins, 2018; Hecksteden et al., 2018; Ross et al., 2019; Atkinson et al., 2019).

In parallel‐arm exercise randomized controlled trials (RCTs), the standard deviation of individual responses (SD_IR_), the amount by which the true effect of the treatment differs between individuals (Hopkins, 2015) (described in detail below), has been forwarded as an appropriate and robust statistical means of quantifying the magnitude of individual differences in training responsiveness (Atkinson and Batterham, 2015). Importantly, there are potential limitations associated with parallel‐arm exercise RCTs that merit consideration when interpreting the SD_IR_. However, despite several exercise training studies utilizing the SD_IR_ (Stock et al., 2016; Williamson et al., 2017; Phillips et al., 2017; Williamson et al., 2018; McLaren et al., 2018; Hammond et al., 2019; Walsh et al., 2019), the potential impact of these limitations have yet to be discussed in detail in the individual response literature.

Thus, the purpose of the current review is to discuss the potential limitations in parallel‐arm exercise RCTs that may limit confidence when interpreting the SD_IR_. It is important to note that this review does not find fault in the mathematical logic underlying the SD_IR_. Further, we agree with previous reports (Atkinson and Batterham, 2015; Williamson et al., 2017; Atkinson et al., 2019) that calculating the SD_IR_ is the only approach for determining whether interindividual variability can be attributed to an effect of exercise *per se* in parallel‐arm exercise RCTs. In this review, we highlight potential external and inherent limitations that may affect the data obtained from parallel‐arm exercise RCTs and consequently limit confident interpretation of the SD_IR_ as an estimate of true individual differences in training responsiveness. Given the recent focus on the application of personalized exercise‐based medicine (Buford et al., 2013; Ross et al., 2019), this review aims to better inform researchers in exercise science about the logic underlying the SD_IR_ and the potential pitfalls associated with parallel‐arm exercise RCTs that may confound its use as an estimate of variability in training responsiveness attributable to exercise.

## Sources of Variation Impacting an Individual’s Observed Response to Training

In this section, we discuss the different sources of variability that influence an individual’s observed value at a single time point (2.1) and observed pre–post change following an intervention (2.2). The terminology used in this section is a synthesis of terms derived from a series of previously published papers (Hopkins, 2000; Hopkins, 2000; Senn, 2001; Hopkins, 2004; Senn et al., 2010; Scharhag‐Rosenberger et al., 2012; Bouchard et al., 2012; Astorino and Schubert, 2014; Leifer et al., 2014; Bentley et al., 2014; Atkinson and Batterham, 2015; Hopkins, 2015; Hecksteden et al., 2015; Arnold et al., 2015; Ross et al., 2015; Raleigh et al., 2016; Gurd et al., 2016; Bonafiglia et al., 2016; Astorino et al., 2016; Senn, 2016; Montero and Lundby, 2017; deLannoy et al., 2017; Williamson et al., 2017; Cadore et al., 2017; Clarke et al., 2017; Alvarez et al., 2017; Williamson et al., 2018; Swinton et al., 2018; Hecksteden et al., 2018). We attempt to use the most common term(s) for each source of variability and provide a list of relevant terms with definitions and alternative names in Table 1.

**Table 1:** Synthesis of terms used in this paper and in the individual response literature.

Term used this paper	Defined on page	Articles using this term	Alternative names used in other articles
Observed value	2	(Hopkins, 2000; Leifer et al., 2014; Swinton et al., 2018)	
True value	2	(Hopkins, 2000; Leifer et al., 2014; Atkinson and Batterham, 2015; Hecksteden et al., 2015; Swinton et al., 2018)	
Typical error	2	(Hopkins, 2000; Hopkins, 2000; Hopkins, 2004; Bentley et al., 2014; Arnold et al., 2015; Raleigh et al., 2016; Gurd et al., 2016; Bonafiglia et al., 2016; Montero and Lundby, 2017; Williamson et al., 2017; Cadore et al., 2017; Alvarez et al., 2017; Alvarez et al., 2017; Swinton et al., 2018; Hecksteden et al., 2018)	Random error/noise (Hopkins, 2000; Hecksteden et al., 2015; Hecksteden et al., 2018)
			Technical error (Bouchard et al., 2012; Ross et al., 2015; deLannoy et al., 2017; Clarke et al., 2017)
			Coefficient of variation (*TE* expressed as a percentage of the mean; (Astorino and Schubert, 2014; Astorino et al., 2016; Hecksteden et al., 2018; Hopkins, 2000; Scharhag‐Rosenberger et al., 2012))
Technical error	2	(Hopkins, 2000; Atkinson and Batterham, 2015)	Measurement error (Hecksteden et al., 2015)
			Instrumentation error (Swinton et al., 2018)
Random day‐to‐day variability	2	Williamson et al. (2017)	Biological error/variability (Hopkins, 2000; Williamson et al., 2017; Swinton et al., 2018)
Observed change/response	3	(Hopkins, 2000; Leifer et al., 2014)	
True change/response	3	(Leifer et al., 2014; Atkinson and Batterham, 2015)	
Within‐subject variability (*Δ*WS)	3	(Hecksteden et al., 2015; Hecksteden et al., 2018)	Biological variability (Swinton et al., 2018)
			Within‐patient error (Senn, 2001; Senn, 2016)
			Random within‐subjects variability (Atkinson and Batterham, 2015; Williamson et al., 2017; Williamson et al., 2018)
Standard deviation of individual response (SD_IR_; *V*ΔTRUE)	4/5	(Atkinson and Batterham, 2015; Hopkins, 2015; Williamson et al., 2017; Williamson et al., 2018; Hecksteden et al., 2018)	Subject‐by‐training interaction (Atkinson and Batterham, 2015; Hecksteden et al., 2015; Williamson et al., 2017)
			Patient‐by‐treatment interaction (Senn, 2001; Senn et al., 2010; Senn, 2016)
			Individual responses; Individual trainability; Individual talent; Training responsiveness (Hecksteden et al., 2015)
			True individual differences (Hopkins, 2000; Atkinson and Batterham, 2015)
Variability in observed responses (SD_EX_; SD_CON_)	5/6	(Leifer et al., 2014; Hopkins, 2015; Hecksteden et al., 2015; Hecksteden et al., 2018)	Standard deviation in changes in interventions or controls (Atkinson and Batterham, 2015; Williamson et al., 2017; Williamson et al., 2018)
			Gross response variability (Hecksteden et al., 2015; Hecksteden et al., 2018)
Minimum clinically important difference (MCID)	7	(Atkinson and Batterham, 2015; Williamson et al., 2017; Williamson et al., 2018)	Smallest worthwhile difference/change (Hopkins, 2004; Hecksteden et al., 2015; Swinton et al., 2018; Hecksteden et al., 2018)

### Typical error of measurement

Whenever a measurement is obtained, the observed value that results is influenced by both the individual’s true value and random measurement error. Random measurement error, or the typical error of measurement (TE), results from a combination of the technical error introduced by equipment and/or experimenter reliability and the random day‐to‐day variability in biological factors capable of altering the measured variable. Biological factors contributing to random day‐to‐day variability include factors that can affect an individual’s mental and/or physical state at the time of testing (e.g. behavioural and environmental factors including circadian rhythm, sleep patterns, diet, exercise, etc.; (Hopkins, 2000; Mann et al., 2014; Hecksteden et al., 2015; Ross et al., 2019; Swinton et al., 2018)). The equation below demonstrates that an individual’s observed value is comprised of both their true value (TRUE) and the TE (Leifer et al., 2014):

(1) ObservedValue=TRUE±TE

**Figure 2:**
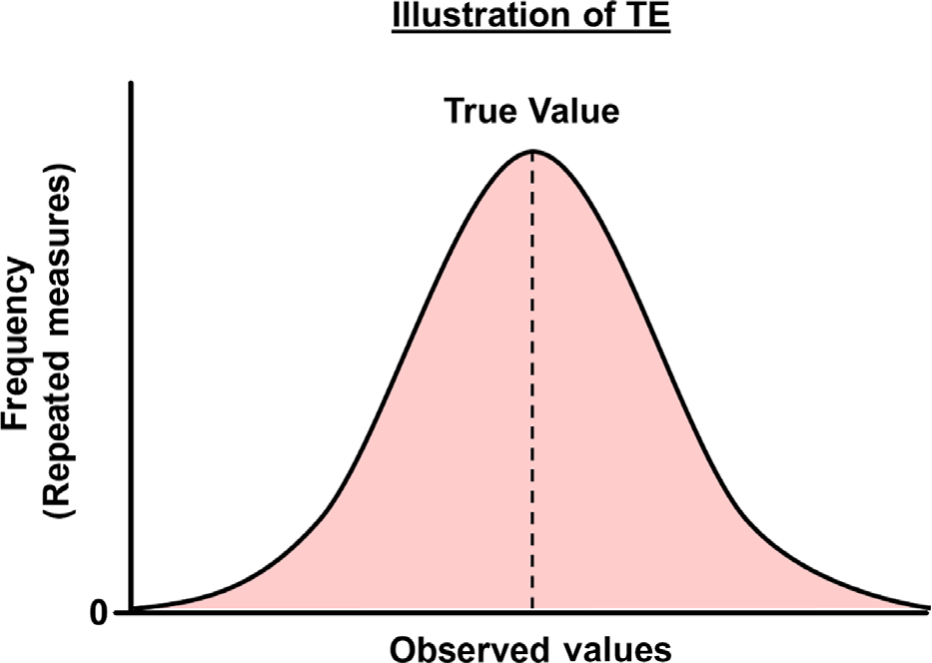
Illustration of the random nature of typical error (*TE*) in the observed values of repeated measures distributed around the true value (vertical dashed line).

Importantly, although both technical error and day‐to‐day biological variability will introduce “noise” into any measurement, this noise is expected to randomly affect the observed value. In other words, the noise introduced by TE will, over the course of repeated measurements, result in observed values that are normally distributed around an individual’s true value (Figure 2). Thus, taking the mean of several measurements at a single time point (e.g. before or after training) will increase the accuracy of the estimate of an individual’s true value (Hopkins, 2004; Hecksteden et al., 2015).

Within the context of a training intervention, an individual’s observed change incorporates both their true change (ΔT) from baseline (PRE) to end of training (POST) and the TE associated with both PRE and POST observed values (ΔTE):

(2) $${\text{Individual}}^{\prime }\text{s}\;\text{Observed}\;\text{Change}=\Delta \text{T}\pm \Delta \text{TE}$$

It is important to emphasize that *TE* (both technical error and day‐to‐day biological variability) would be expected to introduce random noise into both PRE and POST measurements. Thus, while this random noise likely exerts minimal influence on the ability to detect group differences across a training intervention, it can influence an individual’s observed change following training (Hecksteden et al., 2015).

### Within‐subject variability

Biological variability also has the potential to influence an individual’s true change following an exercise training intervention. Chronic changes in behavioral and/or environmental factors external to the prescribed exercise (e.g. changes in long‐term activity patterns or diet quality/quantity; reviewed by (Mann et al., 2014; Solomon, 2018)) can impact an observed change by augmenting or impairing an individual’s true response to an intervention (Senn, 2001; Hecksteden et al., 2015). Because variability in an individual’s mental/physical state could alter their true response to the same exercise intervention administered on different occasions, this source of variability is termed “within‐subject variability” (Table 1; Senn, 2001; Hecksteden et al., 2015). The existence of within‐subject variability requires that ΔT (from equation 2) be further delineated into true changes attributable to exercise (ΔTRUE) and true changes not‐attributable to exercise (i.e., changes attributable to within‐subject variability; ΔWS):

(3) $${\text{Individual}}^{\prime }\text{s}\;\text{Observed}\;\text{Change}=\Delta \text{TRUE}\pm \Delta \text{WS}\pm \Delta \text{TE}$$

Unlike TE, which is expected to have a random effect on observed changes (Figure 2) and remain constant regardless of the duration of an intervention, the impact of ΔWS on an individual’s observed change is expected to increase with longer interventions due to the potential for longer/more substantial behavioral/environmental changes.

Box 1 **Key points from “Sources of Variation Impacting an Individual’s Observed Response to Training” section**

- The “noise” introduced by the typical error of measurement (TE) is expected to randomly affect observed values (Figure 2).
- In addition to TE in both PRE‐ and POST‐intervention measurements, changes in behavioural and/or environmental factors also affect an individual’s observed change to an intervention (termed within‐subject variability).
- Although the influence of TE on an individual’s observed change remains constant regardless of the length of the intervention, the influence of within‐subject variability is expected to increase with longer intervention durations.

## Attempting to Isolate Individual Differences in Training Response: The SD_IR_

Although a repeated cross‐over exercise/control study can theoretically partition the multiple sources of variation that contribute to an individual’s observed change following training (Senn et al., 2010; Hecksteden et al., 2015), this experimental design is costly and time‐consuming. In contrast, estimating the standard deviation of individual responses (SD_IR_) in a parallel‐arm exercise RCT (i.e., one or more experimental arms and one control arm) has been championed as a more feasible approach to isolate the amount by which ΔTRUE differs between individuals (Atkinson and Batterham, 2015; Hopkins, 2015; Atkinson et al., 2019). In this section, we explore how differences in the standard deviations of change scores between the experimental and control arms of a parallel‐arm RCT are used to calculate the SD_IR_. We also highlight the assumptions that permit the SD_IR_ to be interpreted as an estimate of true individual differences in training responsiveness.

### Sources of between‐subject response variability within the exercise arm of an RCT

From this point forward, we will focus on the factors contributing to the variability in observed responses between individuals (i.e., interindividual variability/between‐subject variability in observed responses; Table 1).

Within the exercise arm of a parallel‐arm RCT, the variability in observed responses can be quantified by calculating the standard deviation of the individual change scores (the standard deviation of observed responses to exercise; SD_EX_). Although the variability in the factors contributing to SD_EX_ cannot be isolated for a single arm exercise intervention (Hecksteden et al., 2015), we can theoretically capture these factors using the following equation:

(4) $${\text{SD}}_{\text{EX}}=V\Delta \text{TRUE}\pm V\Delta {\text{WS}}_{\text{EX}}\pm V\Delta {\text{TE}}_{\text{EX}}$$

where *V*ΔTRUE is the between‐subject variability in the true changes attributable to exercise (i.e., the magnitude of true individual differences in training responsiveness), *V*ΔWS_EX_ is the variability in the within‐subject variability within the exercise arm (i.e. the between‐subject variability in true changes not attributable to exercise), and *V*ΔTE_EX_ is the variability in the TE at PRE and POST within the exercise arm.

**Figure 3:**
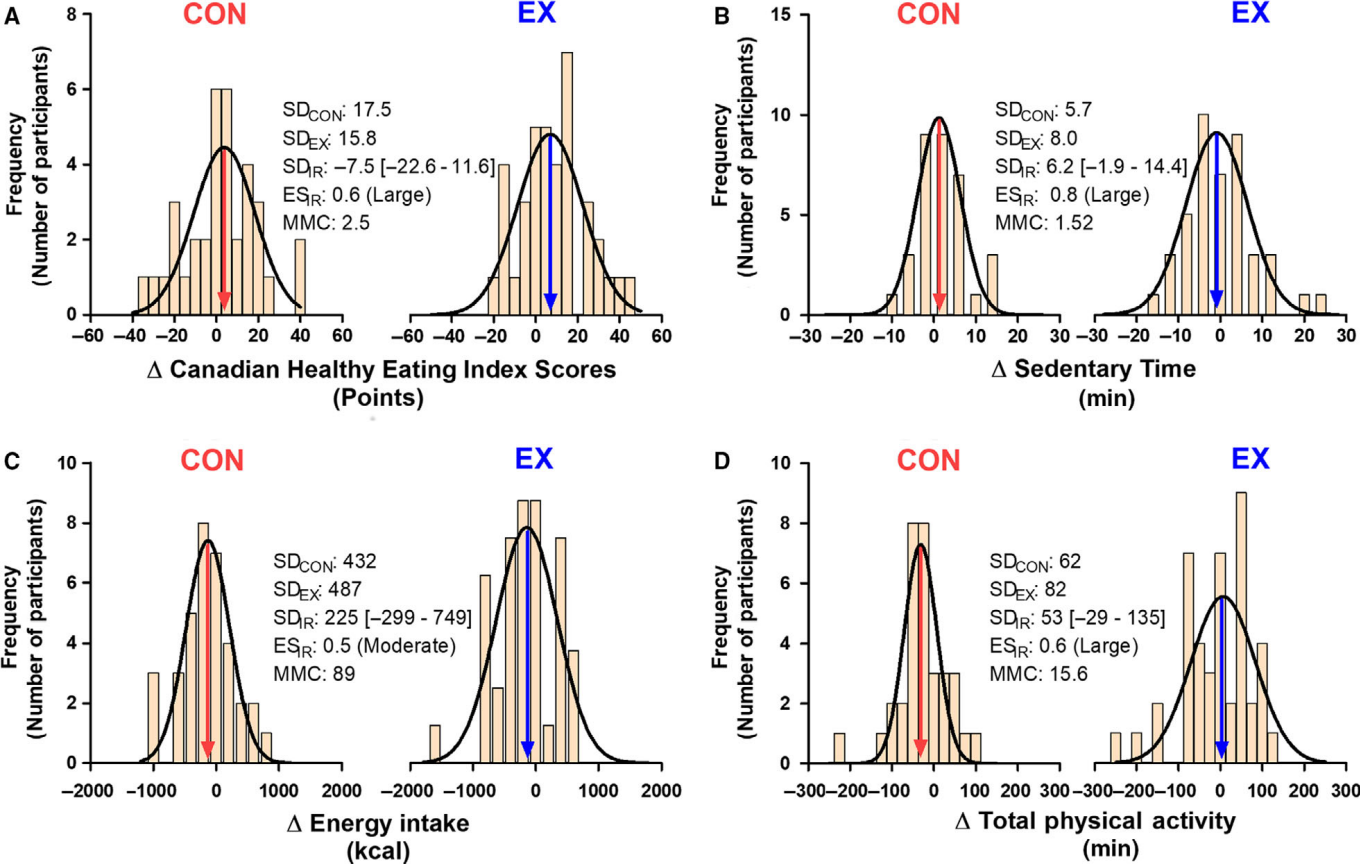
Histograms depicting variability in changes in behavioral factors that are known to influence overall health and fitness following the completion of 24 weeks of exercise training (EX) or a control period (CON). All data were collected from a previously published randomized controlled trial (Ross et al., 2015). Variability in changes in Canadian Healthy Eating Index Scores (A), sedentary time (B), energy intake (C), and total physical activity (D). The EX and CON groups presented in this figure are the same groups presented in Figure 1. See Ross et al. (2013) for more information regarding the measurement of these behavioral outcomes. SD_CON_ and SD_EX_ values represent the variability in observed responses to CON and EX, respectively. SD_IR_ values were calculated using equation 8. Negative SD_IR_ values reflect situations where SD_CON_ exceeded SD_EX_, and SD_IR_ was therefore calculated by switching SD_CON_ and SD_EX_ in equation 8. As recommended by Hopkins (Hopkins, 2015), effect sizes of SD_IR_ values (ES_IR_) were calculated by dividing SD_IR_ values by baseline SD (see Hopkins (2015) for effect size category cut‐points). As previously recommended (Hopkins et al., 2009; Swinton et al., 2018; Hecksteden et al., 2018), minimum meaningful change (MMC) thresholds were determined by multiplying baseline SD by 0.2. The arrows indicate the mean observed response for each behavioral variable.

As with the impact of ΔWS on an individual’s observed response (discussed in “Sources of Variation Impacting an Individual’s Observed Response to Training” section), *V*ΔWS_EX_ reflects variability in changes in behavioral/environmental factors external to the prescribed exercise that can either augment or impair individuals’ true responses (Senn, 2001; Hecksteden et al., 2015). Figure 3 presents variability in changes in behavioral factors in an EX group from a large RCT (Ross et al., 2013; Ross et al., 2015), which potentially demonstrates the existence of *V*ΔWS_EX_ and raises the possibility that variability in these behavioral factors contributed to the SD_EX_ presented in Figure 1. Importantly, the component of variability within SD_EX_ attributed to *V*ΔWS_EX_ and *V*ΔTE_EX_ is purported to occur randomly (Atkinson and Batterham, 2015; Williamson et al., 2017; Williamson et al., 2018). This purported random nature of *V*ΔWS_EX_ has led it to be called “random within‐subjects variability” (Atkinson and Batterham, 2015; Williamson et al., 2017; Williamson et al., 2018). Similar to the effects of ΔTE and ΔWS, the effect of *V*ΔTE_EX_ on SD_EX_ should remain constant regardless of the duration of intervention period while the impact of *V*ΔWS_EX_ on SD_EX_ would be expected to increase with increasing intervention duration.

Because SD_EX_ results from multiple sources of variability, inferences about the existence or magnitude of *V*ΔTRUE cannot be made without quantifying the contributions of *V*ΔWS_EX_ and *V*ΔTE_EX_. As discussed in the next subsection, a control group is needed to estimate the contribution of *V*ΔWS and *V*ΔTE on the variability in observed responses (Atkinson and Batterham, 2015). Thus, attempts to attribute variability in the observed responses to *V*ΔTRUE in single‐arm exercise trials (i.e. lacking a control group) have been justifiably criticized (Atkinson and Batterham, 2015; Williamson et al., 2017).

### Response variability within the control arm of an RCT and calculating SD_IR_

The fundamental assumption inherent to parallel‐arm exercise RCTs is that participants in the treatment and control (CON) groups differ only by the treatment they receive (i.e. standardized exercise training vs. usual care, respectively; (Hopkins, 2018)). Accordingly, it is assumed that the difference between SD_EX_ (see equation 4 above) and the standard deviation of the observed responses to CON (SD_CON_) is the absence of *V*ΔTRUE. Thus, the variability in the observed responses to CON (SD_CON_) can be captured with the following equation:

(5) $${\text{SD}}_{\text{CON}}=V\Delta {\text{WS}}_{\text{CON}}\pm V\Delta {\text{TE}}_{\text{CON}}$$

where *V*ΔWS_CON_ and *V*ΔTE_CON_ are the variability attributable to random within‐subject variability and TE, respectively. Similar to EX, there appears to be variability in changes in behavioral factors in CON (select behavioral factors from a large RCT (Ross et al., 2013; Ross et al., 2015) are presented in Figure 3) and this variability may contribute to SD_CON _(Figure 1A).

If the only difference between EX and CON within a parallel‐arm RCT is the presence (or absence) of exercise, and we assume that variability in within‐subject variability and *TE* are equal between groups (i.e. *V*ΔWS_EX_ = *V*ΔWS_CON_ and *V*ΔTE_EX_ = *V*ΔTE_CON_), subtracting the variability of observed responses to CON (SD_CON_) from the variability in observed responses to EX (SD_EX_) should provide us with an estimate of *V*ΔTRUE as follows:

(6) $${\text{SD}}_{\text{EX}}-{\text{SD}}_{\text{CON}}=\left(V\Delta \text{TRUE}\pm V\Delta {\text{WS}}_{\text{EX}}\pm V\Delta {\text{TE}}_{\text{EX}}\right)-\left(V\Delta {\text{WS}}_{\text{CON}}\pm V\Delta {\text{TE}}_{\text{CON}}\right)$$

wherein *V*ΔWS_EX_ = *V*ΔWS_CON_ and *V*ΔTE_EX_ = *V*ΔTE_CON_; thus, (*V*ΔWS_EX_ *± V*ΔTE_EX_) and (*V*ΔWS_CON_ *± V*ΔTE_CON_) cancel each other out resulting in the following (simplified) equation:

(7) $${\text{SD}}_{\text{EX}}-{\text{SD}}_{\text{CON}}=V\Delta \text{TRUE}$$

The simplification of equation (6) to equation (7) and the underlying logic detailed above provide the foundation for the utility of the SD_IR_ in parallel‐arm exercise RCTs. Specifically, the difference in variability between EX and CON reflects the variability that is attributable to true individual differences in training responsiveness (*V*ΔTRUE). It is important to reiterate that interpreting the SD_IR_ as an estimate of *V*ΔTRUE is based on the assumption that *V*ΔWS and *V*ΔTE are equal between EX and CONs*.* Accordingly, if there is the potential that this assumption is violated, then caution should be applied when interpreting the SD_IR_.

SD_IR_ is calculated using the following equation (Atkinson and Batterham, 2015; Hopkins, 2015; Williamson et al., 2017):

(8) $${\text{SD}}_{\text{IR}}=\sqrt{{\left({\text{SD}}_{\text{EX}}\right)}^{2}-{\left({\text{SD}}_{\text{CON}}\right)}^{2}}$$

Once the SD_IR_ is calculated, confidence intervals and standardized effect sizes can be generated (Hopkins, 2015; Hopkins, 2018) and the magnitude of the SD_IR_ can be interpreted relative to a minimal clinically important difference (MCID) (Atkinson and Batterham, 2015) or a smallest worthwhile change (SWC; typically 0.2 x baseline standard deviation) (Hopkins et al., 2009).

Box 2 **Key points from “Attempting to Isolate Individual Differences in Training Response: The SDIR” section**

- Based on the assumption that typical error (*VΔTE*) and within‐subject variability (*VΔWS*) do not differ between exercise and control arms in an RCT, the SD_IR_ theoretically represents the magnitude of individual differences in training responsiveness (*VΔ*TRUE) (equations (6), (7), (8)).
- If the assumptions of the SD_IR_ are violated, then caution is warranted when interpreting the SD_IR_.

## The Impact of Limitations in Parallel‐Arm Exercise RCT on the Interpretation of the SD_IR_

In “Response variability within the control arm of an RCT and calculating SDIR” section, we discussed that interpreting the SD_IR_ as an estimate of *V*ΔTRUE requires that *V*ΔWS and *V*ΔTE are the same between EX and CON groups (i.e., *V*ΔWS_EX_ = *V*ΔWS_CON_ and *V*ΔTE_EX_ = *V*ΔTE_CON_). In this section, we highlight examples that violate this assumption. Specifically, we highlight external (“External limitations that may affect the interpretation of the SDIR” and “The potential influence of adherence and compliance to the prescribed exercise” sections) and inherent (“Inherent limitations that may affect the interpretation of the SDIR” section) limitations in the design of parallel‐arm exercise RCTs and suggest that these limitations limit confidence when interpreting the SD_IR_ as an estimate of *V*ΔTRUE.

### External limitations that may affect the interpretation of the SD_IR_

As stated in “Attempting to Isolate Individual Differences in Training Response: The SDIR” section, failure to consider SD_CON_ is a major limitation that prevents inference about the existence and/or magnitude of *V*ΔTRUE ( Williamson et al., 2017). Although this section focuses on other external limitations that can occur in RCTs, the issues associated with not considering SD_CON_ are briefly reiterated in the discussion (“Discussion” section) and have been discussed in previous articles (Atkinson and Batterham, 2015; Williamson et al., 2017; Ross et al., 2019; Atkinson et al., 2019).

Even when SD_CON_ is considered, there are external limitations in study design that can occur in parallel‐arm exercise RCTs that may violate the assumption that *V*ΔWS and *V*ΔTE are equal between EX and CON. It is important to acknowledge that these limitations represent deviations from standard guidelines for designing an RCT (Moher et al., 2010). For instance, using different equipment and/or experimenters to measure outcomes in EX vs. CON groups (Phillips et al., 2017) risks introducing differences in *VΔTE* between EX and CON groups. Additionally, study designs that allow for potential between‐group differences in behavioral/environmental factors (e.g., using different durations to separate baseline and follow up measures between EX and CON; collecting EX and CON at different sites (Phillips et al., 2017); etc.) risks introducing differences in *VΔWS* between groups. Non‐optimal RCT designs introduce the possibility that *V*ΔTE_EX_ ≠ *V*ΔTE_CON_ and/or *V*ΔWS_EX_ ≠ *V*ΔWS_CON_ and therefore limit the utility of the SD_IR_ to accurately estimate *V*ΔTRUE (Atkinson et al., 2019).

### The potential influence of adherence and compliance to the prescribed exercise

It is important to note that differences in training adherence (attending the prescribed number of training sessions) and compliance (completing the exercise sessions as prescribed; i.e. achieving the prescribed exercise intensity and/or duration) may also influence the variability in observed responses to exercise training (SD_EX_). This variability would not be attributable to either *VΔ*TRUE or *V*ΔWS_EX_, but would represent an additional source of variance in the observed response to an exercise intervention. We have modified equation 4 to include variability in adherence/compliance to exercise training (*V*ΔAD):

(9) $${\text{SD}}_{\text{EX}}=V\Delta \text{TRUE}\pm V\Delta \text{AD}\pm V\Delta {\text{WS}}_{\text{EX}}\pm V\Delta {\text{TE}}_{\text{EX}}$$

Importantly, variability in participant adherence/compliance to exercise training (*V*ΔAD) further complicates the assumption that EX and CON only differ by *V*ΔTRUE. Specifically, subtracting SD_CON_ from SD_EX_ would not isolate (*V*ΔTRUE) but instead would result in the following (modified based on equation 7; see above):

(10) $${\text{SD}}_{\text{CON}}-{\text{SD}}_{\text{EX}}=V\Delta \text{TRUE}\pm V\Delta \text{AD}$$

The added complexity associated with *V*ΔAD requires that trialists implement a standardized approach that considers participant adherence/compliance prior to calculating the SD_IR_ (e.g., only include data from participants that completed> 90% of supervised training sessions). We refer the reader to published articles that have discussed strategies to account for differences in participant adherence and compliance (Smart et al., 2015; Hecksteden et al., 2018).

### Inherent limitations that may affect the interpretation of the SD_IR_

The impact of the external limitations discussed in “External limitations that may affect the interpretation of the SDIR” and “The potential influence of adherence and compliance to the prescribed exercise” sections can be eliminated, or at least reduced, by performing rigorously designed RCTs. However, even in rigorously controlled exercise RCTs, there may be inherent limitations that threaten the assumption that *VΔTE* and *VΔWS* are random, and thus are equal between EX and CON. Unlike drug trials that administer placebo to the CON group, participants cannot be blinded to their assigned group in exercise RCTs (Smart et al., 2015; Hecksteden et al., 2018). Non‐blinded group assignment risks introducing performance/participant preference bias (Halpern, 2003; Higgins et al., 2011); a type of bias that causes participants to alter their behavior during the course of an intervention based on the knowledge of, and potential preference toward/against, their assigned group (Halpern, 2003). Thus, it is possible that performance/preference bias results in differences in variability in behavioral changes between EX and CON (Figure 3), which violates the assumption that *V*ΔWS is equal between groups.

We have performed two novel analyses in an attempt to determine whether performance/participant preference bias exists in exercise RCTs. First, we synthesized dropout information from several large parallel‐arm exercise RCTs (Table 2). Interestingly, we found that despite similar dropout rates (*P* = 0.9), significantly more (*P* < 0.001) CON participants (12.8% of total sample) dropped out due to dissatisfaction with their group assignment than EX participants (3.4% of total sample; see Table 2). This finding is consistent with the assertion that participants prefer to be assigned to EX over CON (Sluijs et al., 2006; Hertogh et al., 2010; Hecksteden et al., 2018) and raises the possibility that exercise RCTs inherently introduce performance/preference bias that may contribute to differences in *V*ΔWS between groups.

**Table 2:** Reasons for dropouts pooled across six large parallel‐arm exercise randomized controlled trials conducted in middle‐aged, overweight/obese adults free of cardiovascular disease and type 2 diabetes^b^.

	Exercise Group	Control Group
Total Number of Participants	966	288
Reasons for Dropout		
Dissatisfaction with group^a^	33 (3.4%)	37 (12.8%)
No contact	8 (0.8%)	3 (1.0%)
Time commitment	50 (5.2%)	6 (2.1%)
Other	121 (12.5%)	18 (6.3%)
Total Number of Dropouts	212 (21.9%)	64 (22.2%)

We performed 2x2 chi‐squared analyses on the proportion of dropouts (dropouts vs. completers) and the number of participants who dropped out due to dissatisfaction (dropouts due dissatisfaction vs. dropouts not due to dissatisfaction) between EX and CON. References for the six randomized controlled trials: (Ross et al., 2000; Ross et al., 2004; Slentz et al., 2004; Church et al., 2007; Davidson et al., 2009; Ross et al., 2015, b). Percentages are relative to total number of participants within each group.

a Significant difference (*P* < 0.001) between groups.

b This table only includes dropout data from exercise and control groups. Groups that followed dietary interventions without a prescribed exercise intervention were excluded from this analysis.

Next, in an attempt to test the assumption that *V*ΔWS is equal between EX and CON, and to try to understand the impact of non‐blinding/preference bias in exercise RCTs, we compared the variability in changes in select behavioral factors (parameters of physical activity and diet) from a large exercise RCT (Ross et al., 2013; Ross et al., 2015). Interestingly, we found that the variability in these factors differed between EX and CON groups with moderate–large SD_IR_ effect sizes (Figure 3). Although this analysis is preliminary, it highlights the potential impact of non‐blinding on behavioral factors believed to contribute to *V*ΔWS*.*

Collectively, these analyses highlight the potential impact of non‐blinded group assignment in parallel‐arm exercise RCTs on data quality. Specifically, we believe these results suggest that inherent pitfalls associated with exercise RCTs violate the assumption that *V*ΔWS_EX_ = *V*ΔWS_CON_. In an attempt to improve the robustness of the SD_IR _in parallel‐arm exercise RCTs, trialists can use statistical approaches (e.g. outlier removal) to identify participants that may have deviated from the prescribed behaviors. However, it may prove difficult, if not impossible, to measure and account for all sources of *VΔWS* when attempting to calculate and interpret the SD_IR_.

Box 3 **Key points from “The Impact of Limitations in Parallel‐Arm Exercise RCT on the Interpretation of the SDIR” section**

- Parallel‐arm exercise RCTs containing external limitations may deliberately introduce between‐group differences in *V*ΔTE and/or *V*ΔWS, thus violating the assumptions that allow the SD_IR_ to estimate *V*ΔTRUE*.*
- Beyond avoidable external limitations, inherent limitations in parallel‐arm exercise RCTs (e.g. inability to blind participants) also risk violating the assumption that *V*ΔWS is equal between EX and CON.

## Discussion

In the previous section, we discussed that limitations of parallel‐arm exercise RCTs may invalidate the assumption that *VΔTE* and *VΔWS* are equal between EX and CON due to: (1) non‐optimal RCT designs (“External limitations that may affect the interpretation of the SDIR” section), (2) variability in participant adherence/compliance to exercise training (“The potential influence of adherence and compliance to the prescribed exercise” section), and (3) inherent limitations (e.g. inability to blind participants to group assignment; “Inherent limitations that may affect the interpretation of the SDIR” section). Taken together, the previous section suggests that caution is warranted when interpreting the SD_IR_ as an estimate of *V*ΔTRUE in parallel‐arm exercise RCTs.

It is important to note that the above‐mentioned limitations are specific to parallel‐arm exercise RCTs. RCTs that are devoid of these limitations (e.g., drug trials where participants can be blinded) may not violate the assumption that *V*ΔTE and *V*ΔWS are equal between EX and CON. Additionally, although acute exercise studies involve non‐blinded participants, these studies are relatively short (e.g. measurements collected at baseline and three hours–postacute exercise (Egan and Zierath, 2013; Perry and Hawley, 2017)) and may not provide enough time for behavioral–environmental differences (i.e., factors contributing to *V*ΔWS) to emerge between EX and CON. To our knowledge, only one acute exercise study has utilized the SD_IR_ (Bonafiglia et al., 2019), highlighting acute exercise as a feasible model for exploring the existence and magnitude of *V*ΔTRUE. Subsequent to establishing the existence of *V*ΔTRUE, researchers can explore potential mechanisms that contribute to interindividual differences in training responsiveness (see “conceptual framework” in (Atkinson and Batterham, 2015)).

It is also important to reiterate that the majority of previous reports examining individual responses to exercise training have not included a CON group (Hautala et al., 2006; Vollaard et al., 2009; Astorino and Schubert, 2014; Wolpern et al., 2015; Raleigh et al., 2016; Gurd et al., 2016; Bonafiglia et al., 2016; Astorino et al., 2016; Montero and Lundby, 2017) or analyzed SD_CON_ (Sisson et al., 2009; Ross et al., 2015). In the absence of SD_CON_, it is impossible to partition the contributions of *V*ΔTRUE and *V*ΔTE/*V*ΔWS as the counterfactual (i.e., an estimate of what would have happened had a participant in EX been allocated to CON) remains unknown (Williamson et al., 2017). Although we suggest that caution is warranted when interpreting the SD_IR_, failing to consider SD_CON_ represents a larger and more problematic issue in the individual response literature.

## Conclusion and Future Directions

The SD_IR_ statistic estimates whether variability in the observed responses to exercise training can be attributed to an effect of *V*ΔTRUE* per se* (Atkinson and Batterham, 2015). However, external limitations and non‐blinded group assignment may confound the robustness of the SD_IR_. Therefore, we suggest that future studies consider the potential limitations in parallel‐arm exercise RCTs when interpreting the SD_IR_ as an estimate of *V*ΔTRUE.

While the SD_IR _statistic is relevant to parallel‐arm exercise RCTs, there are other statistical approaches that are useful for clinical/applied settings. Specifically, there are several approaches for estimating whether an individual has benefited from an exercise intervention (Hopkins, 2000; Swinton et al., 2018; Hecksteden et al., 2018; Ross et al., 2019; Bonafiglia et al., 2019). Although these approaches are not able to determine why an individual has/has not benefited following an intervention, they provide information that can be used to guide individualized exercise prescription decision‐making (Bonafiglia et al., 2018). Therefore, although the SD_IR_ is the only statistic able to assess the existence/magnitude of *V*ΔTRUE in parallel‐arm exercise RCTs (Atkinson et al., 2019), different statistical approaches (Hopkins, 2000; Swinton et al., 2018; Hecksteden et al., 2018; Ross et al., 2019; Bonafiglia et al., 2019) can be used in future studies that wish to investigate the application of personalized exercise‐based medicine.

## Conflict of Interest

The authors have declared that no conflicts of interests exist.

## Data Availability Statement

The raw data supporting the conclusions of this manuscript will be made available by the authors, without undue reservation, upon request.

## References

[phy214163-bib-0001] Alvarez, C. , R. Ramírez‐Campillo , R. Ramírez‐Vélez , and M. Izquierdo . 2017 Effects of 6‐weeks high‐intensity interval training in schoolchildren with insulin resistance: Influence of biological maturation on metabolic, body composition, cardiovascular and performance non‐responses. Front Physiol 8;444. 28706490 10.3389/fphys.2017.00444 PMC5489677

[phy214163-bib-0002] Alvarez, C. , R. Ramírez‐Campillo , R. Ramírez‐Vélez , and M. Izquierdo . 2017 Prevalence of non‐responders for glucose control markers after 10 weeks of high‐intensity interval training in adult women with higher and lower insulin resistance. Front Physiol. 8:479. 28729841 10.3389/fphys.2017.00479 PMC5498508

[phy214163-bib-0003] Arnold, J. T. , S. J. Oliver , T. M. Lewis‐jones , L. J. Wylie , and J. H. Macdonald . 2015 Beetroot juice does not enhance altitude running performance in well‐trained athletes. Appl. Physiol. Nutr. Metab. 40:590–595. 25942474 10.1139/apnm-2014-0470

[phy214163-bib-0004] Astorino, T. A. , and M. M. Schubert . 2014 Individual responses to completion of short‐term and chronic interval training: a retrospective study. PLoS ONE 9:e97638. 24847797 10.1371/journal.pone.0097638 PMC4029621

[phy214163-bib-0005] Astorino, T. A. , R. M. Edmunds , A. Clark , L. King , R. M. Gallant , S. Namm , et al. 2016 High‐intensity interval training increases cardiac output and VO2max. Med. Sci. Sports Exerc. 49:265–273. 10.1249/MSS.0000000000001099 27669447

[phy214163-bib-0006] Atkinson, G. , and A. M. Batterham . 2015 True and false interindividual differences in the physiological response to an intervention. Exp. Physiol. 100:577–588 Available from http://www.ncbi.nlm.nih.gov/pubmed/25823596 25823596 10.1113/EP085070

[phy214163-bib-0007] Atkinson, G. , P. Williamson , and A. M. Batterham . 2019 Issues in the determination of “responders” and “non‐responders” in physiological research. Exp Physiol.;EP087712 Available from: https://onlinelibrary.wiley.com/doi/abs/10.1113/EP087712 10.1113/EP087712 31116468

[phy214163-bib-0008] Bentley, R. F. , J. M. Kellawan , J. S. Moynes , V. J. Poitras , J. J. Walsh , and M. E. Tschakovsky . 2014 Individual susceptibility to hypoperfusion and reductions in exercise performance when perfusion pressure is reduced: evidence for vasodilator phenotypes. J. Appl. Physiol. 117:392–405 Available from http://jap.physiology.org/cgi/doi/10.1152/japplphysiol.01155.2013 24970851 10.1152/japplphysiol.01155.2013 PMC4137234

[phy214163-bib-0009] Bonafiglia, J. T. , M. P. Rotundo , J. P. Whittall , T. D. Scribbans , R. B. Graham , and B. J. Gurd . 2016 Inter‐individual variability in the adaptive responses to endurance and sprint interval training: A randomized crossover study. PLoS ONE 11:e0167790. 27936084 10.1371/journal.pone.0167790 PMC5147982

[phy214163-bib-0010] Bonafiglia, J. T. , B. A. Edgett , B. L. Baechler , M. W. Nelms , C. A. Simpson , J. Quadrilatero , et al. 2017 Acute upregulation of PGC‐1α mRNA correlates with traininginduced increases in SDH activity in human skeletal muscle. Appl. Physiol. Nutr. Metab. 42:656‐666. 28177701 10.1139/apnm-2016-0463

[phy214163-bib-0011] Bonafiglia, J. T. , M. W. Nelms , N. Preobrazenski , C. LeBlanc , L. Robins , S. Lu , et al. 2018 Moving beyond threshold‐based dichotomous classification to improve the accuracy in classifying non‐responders. Physiol Rep. 6:e13928. 30488594 10.14814/phy2.13928 PMC6429972

[phy214163-bib-0012] Bonafiglia, J. T. , K. J. Menzies , and B. J. Gurd . 2019 Gene expression variability in human skeletal muscle transcriptome responses to acute resistance exercise. Exp. Physiol. Available from: http://doi.wiley.com/10.1113/EP087436 10.1113/EP087436 30758087

[phy214163-bib-0013] Bonafiglia, J. T. , R. Ross , and B. J. Gurd . 2019 The application of repeated testing and monoexponential regressions to classify individual cardiorespiratory fitness responses to exercise training. Eur J Appl Physiol. 119:889–900. 10.1007/s00421-019-04078-w 30666410

[phy214163-bib-0014] Bouchard, C. , P. An , T. Rice , J. S. Skinner , J. H. Wilmore , J. Gagnon , et al. 1999 Familial aggregation of VO2max response to exercise training: results from the HERITAGE Family Study. J Appl Physiol. 87:1003–1008. 10484570 10.1152/jappl.1999.87.3.1003

[phy214163-bib-0015] Bouchard, C. , S. N. Blair , T. S. Church , C. P. Earnest , J. M. Hagberg , K. Häkkinen , et al. 2012 Adverse metabolic response to regular exercise: is it a rare or common occurrence? PLoS ONE 7:e37887. 22666405 10.1371/journal.pone.0037887 PMC3364277

[phy214163-bib-0016] Buford, T. W. , M. D. Roberts , and T. S. Church . 2013 Toward exercise as personalized medicine. Sport Med. 43:157–165. 10.1007/s40279-013-0018-0 PMC3595541 23382011

[phy214163-bib-0017] Cadore, E. L. , R. S. Pinto , J. L. Teodoro , L. X. N. da Silva , E. Menger , C. L. Alberton , et al. 2017 Cardiorespiratory adaptations in elderly men following different concurrent training regimes. J. Nutr. Heal. Aging. 1–8.10.1007/s12603-017-0958-429582887

[phy214163-bib-0018] Church, T. S. , C. P. Earnest , J. S. Skinner , and S. N. Blair . 2007 Effects of different doses of physical activity on cardiorespiratory fitness among sedentary, Overweight or Obese Postmenopausal. JAMA 297:2081–2091. 17507344 10.1001/jama.297.19.2081

[phy214163-bib-0019] Clarke, J. , L. De Lannoy , and R. Ross . 2017 Comparison of measures of maximal and submaximal fitness in response to exercise. Med. Sci. Sports Exerc. 49:711–6. 27870794 10.1249/MSS.0000000000001164

[phy214163-bib-0020] Davidson, L. , R. Hudson , K. Kilpatrick , J. Kuk , K. McMillan , P. Janiszewski , et al. 2009 Effects of exercise modality on insulin resistance and functional limitation in older adults: a randomized controlled trial. Arch. Intern. Med. 169:122–131 Available from http://search.ebscohost.com/login.aspx?direct=true&db=rzh&AN=105634402&site=ehost-live 19171808 10.1001/archinternmed.2008.558

[phy214163-bib-0021] deLannoy, L. , J. Clarke , P. J. Stotz , and R. Ross . 2017 Effects of intensity and amount of exercise on measures of insulin and glucose: Analysis of inter‐individual variability. PLoS ONE 12:e0177095 Available from: http://dx.plos.org/10.1371/journal.pone.0177095 28493912 10.1371/journal.pone.0177095 PMC5426643

[phy214163-bib-0022] Edgett, B. A. , J. T. Bonafiglia , B. L. Baechler , J. Quadrilatero , and B. J. Gurd . 2016 The effect of acute and chronic sprint‐interval training on LRP130, SIRT3, and PGC‐1α expression in human skeletal muscle. Physiol. Rep. 4:e12879. 27604398 10.14814/phy2.12879 PMC5027339

[phy214163-bib-0023] Egan, B. , and J. R. Zierath . 2013 Exercise metabolism and the molecular regulation of skeletal muscle adaptation. Cell Metab 17:162–184. 10.1016/j.cmet.2012.12.012 23395166

[phy214163-bib-0024] Gurd, B. J. , M. D. Giles , J. T. Bonafiglia , J. P. Raleigh , J. C. Boyd , J. K. Ma , et al. 2016 Incidence of nonresponse and individual patterns of response following sprint interval training. Appl. Physiol. Nutr. Metab. 41(3):229–234 Available from http://www.nrcresearchpress.com/doi/10.1139/apnm-2015-0449 26854820 10.1139/apnm-2015-0449

[phy214163-bib-0025] Halpern, S. D. .2003 Evaluating preference effects in partially unblinded, randomized clinical trials. J. Clin. Epidemiol. 56:109–15. 12654404 10.1016/s0895-4356(02)00598-x

[phy214163-bib-0026] Hammond, B. P. , P. J. Stotz , A. M. Brennan , B. Lamarche , A. G. Day , and R. Ross .2019 Individual variability in waist circumference and body weight in response to exercise. Med. Sci. Sports Exerc. 51:315–322. 30216237 10.1249/MSS.0000000000001784

[phy214163-bib-0027] Hautala, A. J. , A. M. Kiviniemi , T. H. Mäkikallio , H. Kinnunen , S. Nissilä , H. V. Huikuri , et al. 2006 Individual differences in the responses to endurance and resistance training. Eur. J. Appl. Physiol. 96:535–542. 16369817 10.1007/s00421-005-0116-2

[phy214163-bib-0028] Hecksteden, A. , J. Kraushaar , F. Scharhag‐Rosenberger , D. Theisen , S. Senn , and T. Meyer .2015 Individual response to exercise training ‐a statistical perspective. J. Appl. Physiol. 118:1450–1459. 25663672 10.1152/japplphysiol.00714.2014

[phy214163-bib-0029] Hecksteden, A. , W. Pitsch , F. Rosenberger , and T. Meyer . 2018 Repeated testing for the assessment of individual response to exercise training. J. Appl. Physiol. 124:1567–1579. Available from http://www.physiology.org/doi/10.1152/japplphysiol.00896.2017 29357481 10.1152/japplphysiol.00896.2017

[phy214163-bib-0030] Hecksteden, A. , O. Faude , T. Meyer , and L. Donath . 2018 How to construct, conduct and analyze an exercise training study? Front Physiol. 9:1–15. 30140237 10.3389/fphys.2018.01007 PMC6094975

[phy214163-bib-0031] Hertogh, E. M. , A. J. Schuit , P. H. M. Peeters , and E. M. Monninkhof . 2010 Noncompliance in lifestyle intervention studies: the instrumental variable method provides insight into the bias. J. Clin. Epidemiol. 63:900–906. 10.1016/j.jclinepi.2009.10.007 20189770

[phy214163-bib-0032] Higgins, J. P. T. , D. G. Altman , P. C. Gøtzsche , P. Jüni , D. Moher , A. D. Oxman , et al. 2011 The Cochrane Collaboration’s tool for assessing risk of bias in randomised trials. BMJ 343:1–9. 10.1136/bmj.d5928 PMC3196245 22008217

[phy214163-bib-0033] Hopkins, W. G . 2000 Measures of reliability in sports medicine and science. Sport Med 30:1–15. 10.2165/00007256-200030010-00001 10907753

[phy214163-bib-0034] Hopkins, W. G. Precision of the estimate of a subject’s true value (Excel spreadsheet). In: a new view of statistics. Internet society for sport science. 2000 p. www.sportsci.org/resource/stats/xprecisionsubject

[phy214163-bib-0035] Hopkins, W. G. 2000 Precision of the estimate of a subject’s true value (Excel spreadsheet). A new view of statistics: internet society for sports science.

[phy214163-bib-0036] Hopkins, W. G . 2004 How to interpret changes in an athletic performance test. Sportscience 8:1–7.

[phy214163-bib-0037] Hopkins, W. G . 2015 Individual responses made easy. J. Appl. Physiol. 118:1444–1446 Available from http://jap.physiology.org/lookup/doi/10.1152/japplphysiol.00098.2015 25678695 10.1152/japplphysiol.00098.2015

[phy214163-bib-0038] Hopkins, W. G . 2018 Design and analysis for studies of individual responses. Sportscience. 22:39–51.

[phy214163-bib-0039] Hopkins, W. G. , S. W. Marshall , A. M. Batterham , and J. Hanin . 2009 Progressive statistics for studies in sports medicine and exercise science. Med. Sci. Sports Exerc. 41:3–12. 19092709 10.1249/MSS.0b013e31818cb278

[phy214163-bib-0040] Leifer, E. S. , C. A. Brawner , J. L. Fleg , W. E. Kraus , D. J. Whellan , and I. L. Piña , et al. 2014 Are there negative responders to exercise training among heart failure patients? Med. Sci. Sports Exerc. 46:219–224. 23860416 10.1249/MSS.0b013e3182a44164 PMC3893314

[phy214163-bib-0041] MacPherson, R. E. K. , T. J. Hazell , T. D. Olver , D. H. Paterson , and P. W. R. Lemon . 2011 Run sprint interval training improves aerobic performance but not maximal cardiac output. Med. Sci. Sports Exerc. 43:115–122. 20473222 10.1249/MSS.0b013e3181e5eacd

[phy214163-bib-0042] Mann, T. N. , R. P. Lamberts , and M. I. Lambert . 2014 High responders and low responders: Factors associated with individual variation in response to standardized training. Sport Med. 44:1113–1124. 10.1007/s40279-014-0197-3 24807838

[phy214163-bib-0043] McLaren, S. J. , A. Smith , J. D. Bartlett , I. R. Spears , and M. Weston . 2018 Differential training loads and individual fitness responses to pre‐season in professional rugby union players. J. Sports Sci. 36:1–9. doi: 10.1080/02640414.2018.1461449 29629620

[phy214163-bib-0044] McPhee, J. S. , A. G. Williams , J. Perez‐Schindler , H. Degens , K. Baar , and D. A. Jones . 2011 Variability in the magnitude of response of metabolic enzymes reveals patterns of co‐ordinated expression following endurance training in women. Exp. Physiol. 96:699–707. 21571817 10.1113/expphysiol.2011.057729

[phy214163-bib-0045] Moher, D. , S. Hopewell , K. F. Schulz , V. Montori , P. C. Gøtzsche , P. J. Devereaux , et al. 2010 CONSORT 2010 explanation and elaboration: updated guidelines for reporting parallel group randomised trials. BMJ 2010:340. 10.1136/bmj.c869 PMC2844943 20332511

[phy214163-bib-0046] Montero, D. , and C. Lundby . 2017 Refuting the myth of non‐response to exercise training: ‘non‐responders’ do respond to higher dose of training. J Physiol. 595:3377–3387. 28133739 10.1113/JP273480 PMC5451738

[phy214163-bib-0047] Perry, C. G. R. , and J. A. Hawley . 2017 Molecular basis of exercise‐induced skeletal muscle mitochondrial biogenesis: historical advances, current knowledge, and future challenges. Cold Spring Harb. Perspect. Med:a029686 Available from: http://perspectivesinmedicine.cshlp.org/lookup/doi/10.1101/cshperspect.a029686 10.1101/cshperspect.a029686 PMC6120690 28507194

[phy214163-bib-0048] Phillips, B. E. , B. M. Kelly , M. Lilja , J. G. Ponce‐González , R. J. Brogan , and D. L. Morris , et al. 2017 A practical and time‐efficient high‐intensity interval training program modifies cardio‐metabolic risk factors in adults with risk factors for type II diabetes. Front. Endocrinol. (Lausanne) 8:229 Available from http://journal.frontiersin.org/article/10.3389/fendo.2017.00229/full 28943861 10.3389/fendo.2017.00229 PMC5596071

[phy214163-bib-0049] Raleigh, J. P. , M. D. Giles , T. D. Scribbans , B. A. Edgett , L. J. Suwula , J. T. Bonafiglia , et al. 2016 The impact of work‐mathced interval training on VO2peak and VO2 kinetics: diminishing returns with increasing intensity. Appl. Physiol. Nutr. Metab. 41:706–713. 27337599 10.1139/apnm-2015-0614

[phy214163-bib-0050] Raleigh, J. P. , M. D. Giles , H. Islam , M. W. Nelms , R. F. Bentley , J. H. Jones , et al. 2018 Contribution of central and peripheral adaptations to changes in VO2max following four weeks of sprint interval training. Appl. Physiol. Nutr. Metab. 43:1059–1068 Available from http://www.nrcresearchpress.com/doi/10.1139/apnm-2017-0864 29733694 10.1139/apnm-2017-0864

[phy214163-bib-0051] Ross, R. , D. Dagnone , P. J. H. Jones , H. Smith , A. Paddags , R. Hudson , et al. 2000 Diet‐induced weight loss or exercise‐induced weight loss in men. Ann. Intern. Med. 133:92–103. 10896648 10.7326/0003-4819-133-2-200007180-00008

[phy214163-bib-0052] Ross, R. , I. Janssen , J. Dawson , A. M. Kungl , J. L. Kuk , S. L. Wong , et al. 2004 Exercise‐induced reduction in obesity and insulin resistance in women: A randomized controlled trial. Obes Res. 12:789–798. 15166299 10.1038/oby.2004.95

[phy214163-bib-0053] Ross, R. , R. Hudson , A. G. Day , and M. Lam . 2013 Dose‐response effects of exercise on abdominal obesity and risk factors for cardiovascular disease in adults: Study rationale, design and methods. Contemp. Clin. Trials 34:155–160. 10.1016/j.cct.2012.10.010 23123790

[phy214163-bib-0054] Ross, R. , L. De Lannoy , and P. J. Stotz . 2015 Separate effects of intensity and amount of exercise on interindividual cardiorespiratory fitness response. Mayo Clin Proc. 90:1506–1514. Available from http://linkinghub.elsevier.com/retrieve/pii/S0025619615006400 26455890 10.1016/j.mayocp.2015.07.024

[phy214163-bib-0055] Ross R., Hudson R., Stotz P. J., and Lam M. 2015 Effects of exercise amount and intensity on abdominal obesity and glucose tolerance in obese adults. Ann. Intern. Med. 162:325 Available from: http://annals.org/article.aspx?doi=10.7326/M14-1189 25732273 10.7326/M14-1189

[phy214163-bib-0056] Ross, R. , B. H. Goodpaster , L. G. Koch , M. A. Sarzynski , W. M. Kohrt , N. M. Johannsen , et al. 2019 Precision exercise medicine: understanding exercise response variability. Br. J. Sport Med. 1–13 Available from http://bjsm.bmj.com/ 10.1136/bjsports-2018-100328 PMC6818669 30862704

[phy214163-bib-0057] Scharhag‐Rosenberger, F. , S. Walitzek , W. Kindermann , and T. Meyer . 2012 Differences in adaptations to 1 year of aerobic endurance training: individual patterns of nonresponse. Scand. J. Med. Sci. Sport. 22:113–8. 10.1111/j.1600-0838.2010.01139.x 20561283

[phy214163-bib-0058] Senn, S. 2001 Individual therapy: New dawn or false dawn? Drug. Inf. J. 35:1479–94.

[phy214163-bib-0059] Senn, S . 2016 Mastering variation: variance components and personalised medicine. Stat Med. 35:966–77. 26415869 10.1002/sim.6739 PMC5054923

[phy214163-bib-0060] Senn, S. , K. Rolfe , and S. A. Julious . 2010 Investigating variability in patient response to treatment–a case study from a replicate cross‐over study. Stat. Methods Med. Res. 00:1–11 Available from http://www.ncbi.nlm.nih.gov/pubmed/20739334 10.1177/0962280210379174 20739334

[phy214163-bib-0061] Sisson, S. B. , P. T. Katzmarzyk , C. P. Earnest , C. Bouchard , S. N. Blair , and T. S. Church . 2009 Volume of exercise and fitness nonresponse in sedentary, postmenopausal women. Med. Sci. Sports Exerc. 41:539–545. 19204597 10.1249/MSS.0b013e3181896c4e PMC2669311

[phy214163-bib-0062] Slentz, C. A. , B. D. Duscha , J. L. Johnson , K. Ketchum , L. B. Aiken , and G. P. Samsa , et al. 2004 Effects of the amount of exercise on body weight, body composition, and measures of central obesity. Arch. Intern. Med. 164:31 http://archinte.jamanetwork.com/article.aspx?doi=10.1001/archinte.164.1.31 14718319 10.1001/archinte.164.1.31

[phy214163-bib-0063] Van Sluijs, E. M. F. , M. N. M. Van Poppel , J. W. R. Twisk , and W. Van Mechelen .2006 Physical activity measurements affected participants’ behavior in a randomized controlled trial. J. Clin. Epidemiol. 59:404–11. 16549263 10.1016/j.jclinepi.2005.08.016

[phy214163-bib-0064] Smart, N. A. , M. Waldron , H. Ismail , F. Giallauria , C. Vigorito , V. Cornelissen , et al. 2015 Validation of a new tool for the assessment of study quality and reporting in exercise training studies: TESTEX. Int. J. Evid. Based Healthc. 13:9–18. 25734864 10.1097/XEB.0000000000000020

[phy214163-bib-0065] Solomon, T. P. J . 2018 Sources of inter‐individual variability in the therapeutic response of blood glucose control to exercise in type 2 diabetes: going beyond exercise dose. Front. Physiol. 9:1–17 Available from https://www.frontiersin.org/article/10.3389/fphys.2018.00896/full 30061841 10.3389/fphys.2018.00896 PMC6055062

[phy214163-bib-0066] Stock, M. S. , K. D. Olinghouse , A. S. Drusch , J. A. Mota , J. M. Hernandez , and C. C. Akalonu , et al. 2016 Evidence of muscular adaptations within four weeks of barbell training in women. Hum Mov Sci 45:7–22. doi: 10.1016/j.humov.2015.11.004 26583966

[phy214163-bib-0067] Swinton, P. A. , B. S. Hemingway , B. Saunders , B. Gualano , and E. Dolan . 2018 A Statistical Framework to Interpret Individual Response to Intervention: Paving the way for personalised nutrition and exercise prescription. Front. Nutr. 5:41 Available from: https://www.frontiersin.org/articles/10.3389/fnut.2018.00041/abstract 29892599 10.3389/fnut.2018.00041 PMC5985399

[phy214163-bib-0068] Vollaard, N. B. J. , D. Constantin‐Teodosiu , K. Fredriksson , O. Rooyackers , E. Jansson , P. L. Greenhaff , et al.2009 Systematic analysis of adaptations in aerobic capacity and submaximal energy metabolism provides a unique insight into determinants of human aerobic performance. J. Appl. Physiol. 106:1479–1486. 19196912 10.1152/japplphysiol.91453.2008

[phy214163-bib-0069] Walsh, J. J. , J. T. Bonafiglia , G. S. Goldfield , R. J. Sigal , G. P. Kenny , S. Doucette , et al. 2019 Interindividual variability and individual responses to exercise training in adolescents with obesity. Appl Physiol Nutr Metab. 10.1139/apnm-2019-0088 31121100

[phy214163-bib-0070] Williamson, P. J. , G. Atkinson , and A. M. Batterham . 2017 Inter‐individual responses of maximal oxygen uptake to exercise training: a critical review. Sport Med. 47:1501–1513. Available from http://link.springer.com/10.1007/s40279-017-0680-8 10.1007/s40279-017-0680-8 28097487

[phy214163-bib-0071] Williamson, P. J. , G. Atkinson , and A. M. Batterham . 2018 Inter‐individual differences in weight change following exercise interventions : a systematic review and meta‐ analysis of randomized controlled trials. Obes Rev. 1–16. 10.1111/obr.12682 29701297

[phy214163-bib-0072] Wolpern, A. E. , D. J. Burgos , J. M. Janot , and L. C. Dalleck . 2015 Is a threshold‐based model a superior method to the relative percent concept for establishing individual exercise intensity? a randomized controlled trial. BMC Sports Sci Med Rehabil. 7:16 Available from: http://www.biomedcentral.com/2052-1847/7/16 26146564 10.1186/s13102-015-0011-z PMC4491229

